# Immune repertoire profiling and T cell dysregulation in Peripheral Blood Mononuclear Cells of Type 2 diabetic patients

**DOI:** 10.1371/journal.pone.0332736

**Published:** 2025-10-09

**Authors:** Zheng Bi, Fanjing Wang, Shengmao Wang, Zhaohui Fang

**Affiliations:** 1 Department of Endocrinology, The First Affiliated Hospital of Anhui University of Traditional Chinese Medicine, Hefei, Anhui, People’s Republic of China; 2 Graduate School, Anhui University of Traditional Chinese Medicine, Hefei, Anhui, People’s Republic of China; University of Pittsburgh, UNITED STATES OF AMERICA

## Abstract

**Objective:**

The prevalence of diabetes mellitus (DM) is escalating globally, presenting a significant public health challenge. The immune system, particularly T cells, plays a crucial role in the pathogenesis of diabetes. This study aims to elucidate the characteristics of T cell receptors (TCRs) and immune dysregulation within peripheral blood mononuclear cells (PBMCs) of diabetic patients, with exploratory analysis of microbial profiles.

**Methods:**

We employed high-throughput RNA-seq to analyze the protein-coding genes expression, and function enrichment with different expression, BCR/TCR repertoires and microbial communities in PBMC samples collected from both diabetic patients and healthy controls. Comparative analysis was conducted to identify distinct TCR signatures associated with diabetes. Microbial communities were secondarily assessed via unmapped RNA-seq reads.

**Results:**

Overall, we found different patterns of gene expression, gene function, immune cell proportion, immune repertoire and microbiome between the different DM and control groups. 1145 upregulated 400 down-regulated genes were identified, and immune response function terms were enriched, such as, cell-cell adhesion via plasma-membrane adhesion molecules, and homophilic cell adhesion via plasma membrane adhesion molecules (BP); as well as in the T cell receptor complex, plasma membrane signaling receptor complex, alpha-beta T cell receptor complex (CC), and in antigen binding and immunoglobulin receptor binding (MF). Furthermore, reactome pathway enrichment analysis revealed enrichment of these DEGs in Viral mRNA Translation, Influenza Viral RNA Transcription and Replication, SARS-CoV-1 modulates host translation machinery, Interleukin-6 family signaling, etc. DM PBMC showed significantly lower chao1 index of TCR (including TCRA and TCRB) and reduced expression of TRAV/TRBV genes compared to controls. Enriched pathways included T cell receptor complex, antigen binding, and interleukin-6 signaling. Exploratory analysis of microbial reads revealed decreased alpha diversity (chao1/ACE) in DM and 123 altered taxa, though microbial abundance was low.

**Conclusion:**

Our study provides novel insights into T cell receptor dysregulation in diabetes. The role of PBMC-associated microbiota requires further validation.

## Introduction

Diabetes mellitus (DM) is a chronic metabolic disorder characterized by sustained hyperglycemia (fasting or postprandial). It is now recognized as a life-threatening condition due to its severe complications. These complications encompass acute metabolic derangements, infections, and chronic organ damage, including retinopathy, macroangiopathy, neuropathy, nephropathy, and infertility, all of which require comprehensive clinical management [1,2]. DM represents a major global health crisis. Current data indicate a global prevalence exceeding 537 million, with particularly high rates among the elderly (≈20%) and an incidence of 8.3 per 1,000 individuals [3]. The World Health Organization (WHO) projects this burden will escalate further, driven by urbanization, sedentary lifestyle, unhealthy dietary patterns, and aging population [4,5].

Etiologically, DM arises from complex interactions between genetic susceptibility, environmental triggers, and behavioral factors. Specific gene variants may increase disease risk, while infections or toxin exposures can contribute to its onset [6]. Pathogenically, type 1 diabetes (T1D) involves autoimmune destruction of pancreatic β-cells, whereas type 2 diabetes (T2D) is defined by insulin resistance coupled with progressive β-cell dysfunction [7]. Environmental factors like bacteria, viruses, and fungi influence diabetes susceptibility, while diabetes-related genes also alter gut bacteria composition. Certain gut bacteria are linked to type 2 diabetes (T2D), potentially affecting metabolism and insulin resistance. Microbes in the pancreas may also impact insulin secretion and immune responses. Beyond the gut and pancreas, microbial changes in organs like the liver and kidney may increase T2D risk [8,9]. While microbiota studies in diabetes primarily focus on gut/pancreas, the potential presence of microbial components in circulation remains underexplored. Here, we first characterize TCR repertoire dysregulation in PBMCs of T2D patients, with secondary meta-transcriptomic analysis of microbial reads as a pilot investigation.

RNA-seq (RNA-seq) provides a high-resolution platform for profiling global gene expression, making it a powerful tool for investigating complex diseases such as T2D [10]. Previous studies have leveraged RNA-seq to identify candidate biomarker genes (e.g., CNOT6L, CNOT6, SERPING1, ANPEP) and elucidate molecular pathways underlying T2D pathogenesis [11–13] Additionally, bulk RNA-seq enables comprehensive analysis of immune cell receptors [14,15] and metatrancriptome [16].

Here, we conducted a meta-transcriptomc study of peripheral blood mononuclear cells (PBMCs) in DM patients. To our knowledge, this is the first report characterizing taxonomic shifts in the PBMC meta-transcriptome of DM patients. We further demonstrate the interactions between candidate viral markers and differentially expressed human genes (DEGs), linking these DEGs to disruptions in cellular and metabolic pathways. Our findings support the dysregulation of the PBMC meta-transcriptome in DM patients, suggesting its potential utility as a diagnostic marker.

## Materials and methods

### Preprocessing RNA-seq data

Firstly, the raw fastq.gz of PRJNA914870 were download, which including 50 healthy and 143 T2DM patients, which provides only group labels without clinical metadata. This limitation may affect generalizability..The downloaded raw FASTQ was trimmed and removed technical duplicates using fastp [17]. And the ribosomal RNA reads also removed by hisat2 [18].

### Kallisto pseudo-alignment and differential expression analysis

Transcript-level quantification of protein-coding genes was performed using Kallisto (version 0.46.2) [19], a transcriptome index was first generated from GENCODE cDNA sequences (gencode.v46.pc_transcripts.fa.gz) [20]. Subsequently, Kallisto pseudoalignment was applied to quantify transcript abundances Gene-level expression values (counts and TPM) were derived using tximport [21]. Differential expression analysis was conducted on transcript counts with DESeq2 package [22] for R [23], the genes of which log_1.2_FC ≤ −1 and q < 0.05 were considered as down-DEGs, while log_1.2_FC ≥ 1 and q < 0.05 were considered as down-DEGs.

### CIBERSORT analysis

Cell-type identification by estimating Relative Subsets of RNA Transcripts (CIBERSORT), developed by Newman et al., is a computational method that estimates the abundance of specific cell types within mixed cell populations using gene expression profiles [24]. The CIBERSORT algorithm employs a signature matrix (LM22), comprising 547 genes that distinguish 22 human hematopoietic cell phenotypes. For samples from dataset PRJNA914870, immune cell profiles were generated using CIBERSORT with default parameters. Based on the LM22 output, the total fractions of lymphocytes, B cell, T cell, NK cell, Macrophages, Dendritic cells and Mast cells were aggregated for each sample. Differences in immune cell abundance between groups were assessed using the wilcox.test, with a significance threshold of P-value ≤0.05. Pairwise correlations among immune cells types were evaluated via Spearman's rank correlation analysis.

### IG and TCR data extraction and diversity analysis

VDJ region extraction for IG (IGH, IGK, and IGL), and TCR (TRA, TRB, TRD, and TRG) genes was performed using Mixcr [25] with paired-end RNA-seq FASTQ files as input. Sample SRR22891101 was excluded from subsequent analyses. Diversity analysis and other downstream assessments were conducted using immunarch [26].

### Pathogen read detection and alpha and beta diversity analysis

Human reference genome alignment was performed using STAR [27]. Reads mapped to the human genome were removed, while unmapped reads were retained for downstream microbial analysis. To eliminate potential contaminants (e.g., bacterial, archaeal, viral, or human sequences), we applied Kraken2-based decontamination. Alpha diversity is used to assess the taxonomic diversity within a sample, and multi-index including chao1, Shannon, etc [28,29], were employed. Beta diversity was calculated through the Bray–Curtis dissimilarity matrix [29–31]. Bray-curtis dissimilarity is a statistical method that is used to measure the degree of compositional dissimilarity between two different locations based on the count of each element present [32,33]. Both alpha and beta diversity was calculated using Microeco [34]. The differential test result is stored in the object$res_diff, and LEfSe test was employed to identify the markers of taxa [35].

## Results

### Distinct dynamic transcriptome in PBMC with DM

**Fig 1:**
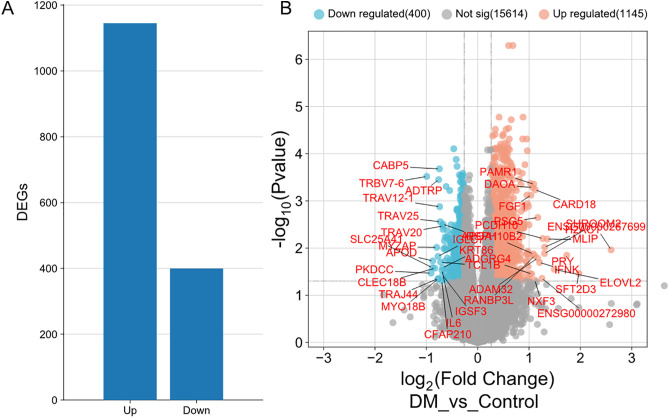
Differential Gene Expression in PBMCs of T2D Patients. (A) Bar plot showing the number of significantly upregulated (1145) and downregulated (400) genes in T2D patients compared to healthy controls. (B) Volcano plot visualizing differential gene expression. Red dots: upregulated genes; blue dots: downregulated genes; gray dots: non-significant genes. Dashed lines indicate significance thresholds.

The PCA plot shows partial but discernible separation between control and diabetic (DM) PBMC samples along the first two principal components (Dim1: 19.5%, Dim2: 16.6%), suggesting moderate transcriptomic differences between groups (Fig 1A). The DEGs were identified between DM and control groups with PBMC by DESeq2. A total of 1545 DEGs were identified, with 1145 up-regulated and 400 down-regulated. And the results were shown with histogram plot (Fig 1B), volcano plot (Fig 1C).

### Enriched pathway of the dynamic transcriptome in PBMC from diabetic patients

**Fig 2:**
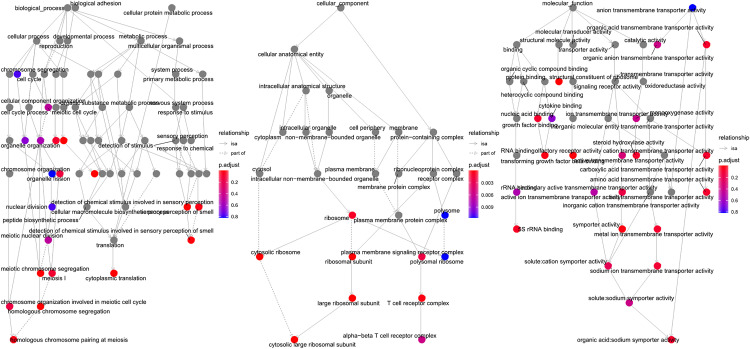
Functional Enrichment Analysis of DEGs Significantly enriched pathways for 1545 DEGs: Top panels: GO enrichment in Biological Process (BP), Cellular Component (CC), and Molecular Function (MF) terms. Highlighted pathways: Viral mRNA Translation, Interleukin-6 family signaling, and SARS-CoV-1 modulates host translation machinery.

The 1,545 DEGs were subjected to functional enrichment analyses using the R package clusterProfiler. Significant enrichment was observed in 392 BP terms, 59 CC terms, and 96 MF terms were significant enrichment (p-value < 0.05). Specifically, GO enrichment analysis revealed that DEGs were significantly enriched in the immune response processes such as, cell-cell adhesion via plasma-membrane adhesion molecules, and homophilic cell adhesion via plasma membrane adhesion molecules (BP); as well as in the T cell receptor complex, plasma membrane signaling receptor complex, and alpha-beta T cell receptor complex (CC). For MF terms, significant enrichment occurred in antigen binding and immunoglobulin receptor binding (Fig 2). The pathway enrichment analysis across the KEGG, reactome and WP database further identified 39, 89 and 19 terms were were significant enrichment, respectively. Notably, Reactome pathways highlighted the involvement of DEGs in Viral mRNA Translation, Influenza Viral RNA Transcription and Replication, SARS-CoV-1 modulates host translation machinery, Interleukin-6 family signaling, etc.

### Immune cell composition and correlation in PMBC from diabetic patients

**Fig 3:**
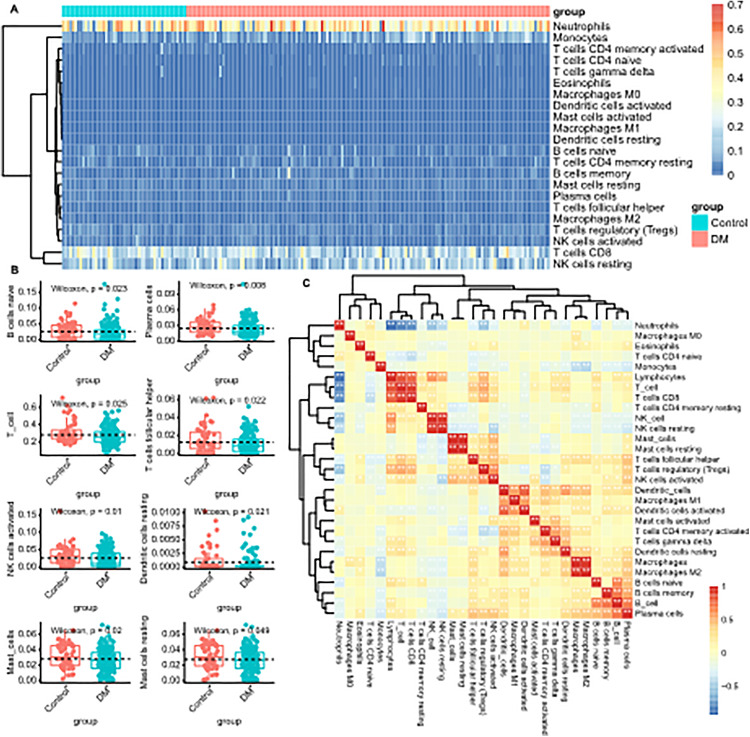
Immune Cell Composition and Correlations in PBMCs: (A) Stacked bar plot of CIBERSORT-derived immune cell proportions (22 subtypes) in T2D vs. healthy groups; (B) Box plots showing significant differences (p < 0.05) in specific cell types; (C) Correlation heatmap of immune cell types. Red: positive correlation; blue: negative correlation.

Following CIBERSORT analysis to estimate the fractions of 22 immune cell types in PBMC of DM and healthy, significant differences in relative immune cell composition were observed in the DM group compared to healthy PBMC. Additionally, the total proportions of B cells, T cells, NK cells, macrophages, and mast cells were calculated (Fig 3A and S1 Fig). Cell abundance, a quantitative measure based on the gene expression profiles, revealed specific alterations in the DM group,.including changes in naive B cells, plasma cells, total T cells, T cells follicular helper, T cells regulatory (Tregs), NK cells activated, Dendritic cells resting, total mast cells and resting mast cells (p < 0.05) (Fig 3B).

Fig 3C presents a correlation heatmap depicting Pearson correlation values for pairwise comparisons among immune cells in CIBERSORT-analyzed samples. As illustrated in Fig 3C, plasma cells exhibited positive Pearson correlations with total B cell, memory B cells, naive B cells, follicular helper T cells (Tfh), and activated NK cells, while showing negative correlations with monocytes and resting NK cells. Total T cell showed positive correlations with CD8^+^ T cells, lymphocytes, regulatory T cells (Tregs), but negative correlations with neutrophils and naive CD4^+^ T cells. Follicular helper T cells demonstrated positive correlations with Tregs, total T cell, plasma cells and activated NK cells, alongside a negative correlation with resting NK cells.

### Immune repertoire profiling and diversity analysis in diabetic PBMCs

Considering that immune-related function and immune cell composition in DM PBMC was different from control group, we employed MiXCR to extract immune receptor sequences from RNA-seq data, assigning them to their respective receptor types: IGH, IGK, and IGL (B cell receptors, BCRs), and TRA, TRB, TRD, and TRG (T cell receptors, TCRs). From 192 subjects, we obtained a total of 1,368,538 reads (297,827 TCR and 1,070,711 BCR) and 706,409 clones (166,620 TCR and 539,789 BCR).

**Fig 4:**
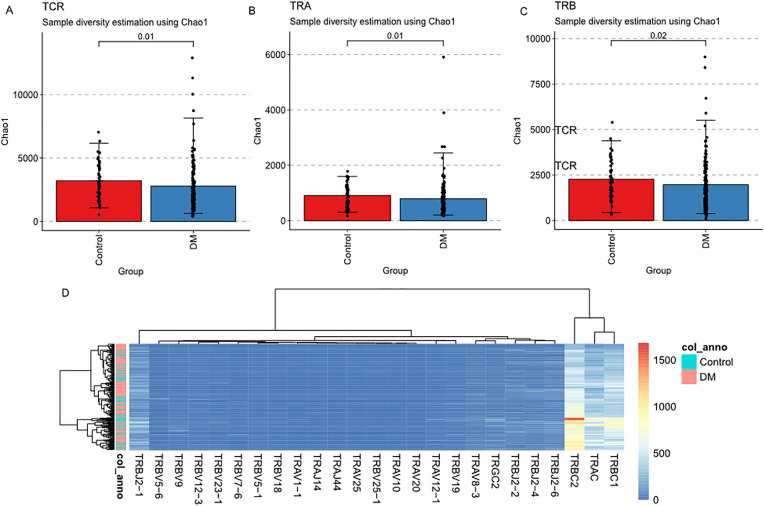
TCR Repertoire Diversity in T2D. Box plots comparing TCR diversity indices between T2D and controls: (A-C) TCR/TRB/TRA chao1 index; (D) Heatmap of Differential expression of TCR genes.

TCR diversity was assessed using multiple metrics to evaluate both the overall repertoire size and the relative abundance of each clonotype. Additionally, we analyzed the diversity characteristics of IGH, IGK, IGL, TRA, TRB, TRD, and TRG using standard indices: chao1 index (number of species in a population), hill index (unified family of diversity indices), true div index (effective number of types), Simpson index (probability of interspecific encounter), and inverse Simpson index, and gini index (inequality among values of a frequency distribution). As shown in Fig 4, the Chao1 index of TCRs was significantly lower in the DM group than in the controls (p = 0.01). However, no significant differences were observed in other TCR diversity indices between the DM and control groups. Specifically, significant reductions in the Chao1 index were detected for TRA and TRB in the DM group (Fig 4). Although the number of TRD clonetypes was relatively low, multiple diversity indices differed significantly between DM and controls. In contrast, no significant differences were found in the diversity indices of IGH, IGK, IGL, TRA, or TRB (S2 Fig).

The comparison of C, V, D, and J gene fragments in TCR and BCR among the two groups. The heatmap in Fig 3C illustrates differential expression of C, V, D, and J gene fragments in T cell receptors (TCR) and B cell receptors (BCR) between diabetic (DM) and control groups. Notably, no significant differences were observed in BCR gene fragments. In contrast, TCR analysis revealed significant reductions in C gene expression in the DM group, including TRAC, TRBC1, TRBC2, and TRGC2. Further analysis identified differentially expressed TCR V and J genes, including TRAV1 − 1, TRAV8 − 3, TRAV10, TRAV12 − 1, TRAV20 and TRAV25, and TRAJ genes, including TRAJ14 and TRAJ44. For TRB, nine V genes including TRBV5 − 1, TRBV5 − 6, TRBV7 − 6, TRBV9, TRBV12 − 3, TRBV18, TRBV19, TRBV23 − 1, and TRBV25 − 1, and four J genes including TRBJ2 − 1, TRBJ2 − 2, TRBJ2 − 4, and TRBJ2 − 6, were significantly differentially expressed between DM and control group.

### Exploratory analysis of microbial reads in PBMC

Given the emerging interest in microbial-host interactions, we performed taxonomic classification on unmapped RNA-seq reads by Kraken2. In PBMC of healthy normal and DM, we observed the presence of 4 predominant phyla of bacteria, led by Proteobacteria (68.24% of total bacteria and 72.24% in healthy and DM), while Actinobacteria (19.72% and 19.72%), Firmicutes (7.03% and 6.06%) and Bacteroidetes (2.23% and 1.97) and Cyanobacteria (1.27% and 1.14%) follow. Devosia (11.23% and 9.37%) and Sphingomonas (5.64% and 6.75%) and Pseudomonas (4.78% and 6.06%) were the most abundant on the level of genus.

**Fig 5:**
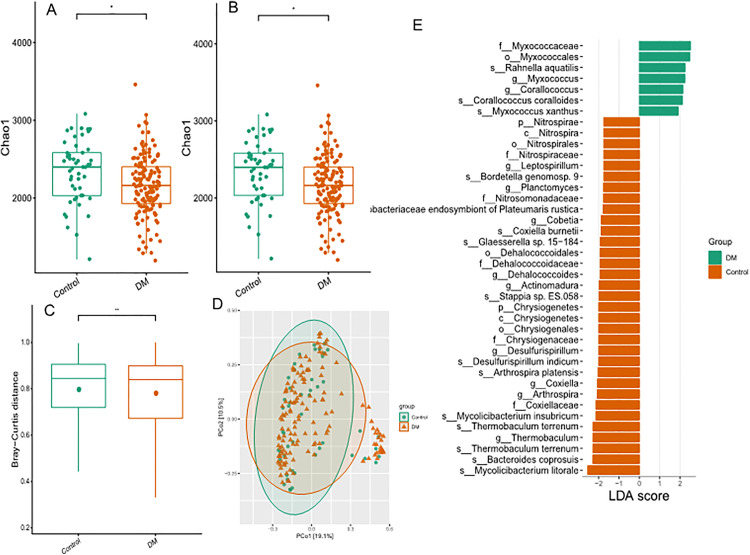
Exploratory Analysis of Microbial Reads in PBMCs. (A) Alpha diversity indices (Chao1, ACE) showing reduced microbial diversity in T2D (p < 0.05); (B) Taxonomic composition at phylum/genus levels; (C) LEfSe analysis identifying 123 differentially abundant taxa. Enriched in controls: Elizabethkingia, Bordetella petrii; enriched in T2D: Lactobacillus crispatus, Microterricola.

The total count of bacteria or viruses was no significant difference between DM and healthy. The characteristics of the diversity of microbiome were analyzed using common diversity indexes. The chao1 index and ACE index was significantly reduced with DM compared to control group (Fig 5A). And distance matrix in beta diversity.

Using the LEfSe function in conjunction with micromicro statistical analysis, we found tha 123 taxa which transcript numbers correlate with disease. The most significantly overrepresented genus in healthy PBMC were Elizabethkingia, Desulfonema, Halanaerobium, Draconibacterium, and overrepresented species include Janthinobacterium sp. 1_2014MBL_MicDiv, Bordetella petrii, Acinetobacter lwoffii, Janthinobacterium sp., Nostoc carneum, Paraburkholderia caffeinilytica, Streptomyces sp. SUK 48, Acinetobacter johnsonii. Meanwhile, four species (Actinoalloteichus sp. GBA129 − 24, Lactobacillus crispatus, Lactobacillus johnsonii and Microterricola viridarii), and two genus (Microterricola and Actinomyces), family Pseudonocardiaceae, and order Pseudonocardiales were overrepresented in DM PBMC. Due to technical limitations of RNA-seq for low-biomass samples, these findings require validation by targeted methods (e.g., 16S rRNA sequencing).

## Discussion

In the present study, we used RNA-Seq, analyzed by functional pathway analyses, MiXCR and kraken2, to identify immune repertoire profiling and microbiome features with DM PBMC. We found impaired T cell-associated signaling, altered plasma membrane signaling, and dyregulated nine types of immune cell, decreased diversity of TCR with gene fragments different expression among DM patients and altered microbiome.

In animal models and humans, the microbiota (referring to bacteria, viruses, fungi, protozoa and archaea) has been identified as an important susceptibility regulator in the development of type 1 diabetes [36]. Viruses, including Coxsackie virus and rotavirus are associated with the development of T1D because 1) they are associated with the development of autoantibodies [37,38], which are predictive biomarkers of immune progression and T1D development [39]; 2) Viral proteins such as enterovirus capsid protein vp1 can be identified in islets [38–42]; 3) The susceptibility of animal models to T1D can be modulated by viral infection [43–47]; 4) Oral rotavirus vaccines have shown potential to protect individuals at risk of developing T1D from the future development of the disease [48]. A large number of literatures have reported the relationship between gut microbes and diabetes. We recently demonstrated that norovirus infection in NOD mice modulates susceptibility to T1D through changes in gut microbiota [49], underscoring the need to increase understanding of broader microbial community interactions. But, there is no study report the microbiota function in PBMC with DM, so this study encode the microbiota profile in PBMC by metatranscriptome strategy. We secondarily observed reduced microbial alpha diversity in DM PBMCs, though the biological significance remains unclear due to low microbial biomass and potential environmental contamination. Very few viral reads were identified, and we focused on bacteria and archaea that we found diversity with DM (with chao1 index and ACE index) was significantly reduced and panel of microbiota altered such as genus Elizabethkingia, Desulfonema, Halanaerobium, Draconibacterium were depleted and Microterricola and Actinomyces were overrepresented in DM. The detection of soil-associated taxa (e.g., Microterricola) may reflect either true biological translocation via the gut or technical artifacts from low-biomass samples. Given the constraints of public data reanalysis, experimental validation is needed to confirm their physiological relevance. While gut/pancreatic microbiota are implicated in diabetes, PBMC-associated microbes may reflect transient bacteremia rather than disease drivers. Future studies with contamination-controlled protocols are needed. Microbial findings are hypothesis-generating; their detection via RNA-seq in PBMCs may include reagent/environmental contaminants and requires orthogonal validation.

There are approximately 10^8^−10^10^ unique TCR clones in adults [50–53]. The capacity of the TCR repertoire can represent the ability to respond to different antigens, and the diversity of the TCR library in the blood can be examined to determine whether it is related to immune status. For example, having a diverse TCR repertoires is associated with an ideal response to cancer immunotherapy [54–56]. It has been reported that compared with non-T1D patients, the diversity of TCR spectrum in peripheral blood of T1D patients is lower [57], and our study found the similar result that TCR, TRA and TRB were significant depletion with DM. Otherwise, we identified a series of difference gene fragments in TCR among the two groups including four C gene, six TRAV genes, two TRAJ, nine TRBV, and four TRBJ2, and almost all genes were depleted with DM. Our data demonstrate that dysregulation in key T cell pathways – specifically IL-6 signaling and TCR complex organization mechanistically supports the paradigm of T cell dysfunction in diabetes.

There are some limitations to our study that should be noted. First, the patient population examined in this study was small. Therefore, surveying different populations with a larger sample size would help confirm our results. Second, we used a lot of RNA-Seq in this study, and future scRNA-Seq and functional experiments will be validated at the cellular level. Owing to the absence of metadata in this dataset, we could not adjust for confounders or disease dynamics—a known challenge in public data reuse. We propose that future studies: (a) prioritize datasets with comprehensive metadata; (b) establish prospective cohorts with standardized clinical recording. This study focused exclusively on Type 2 diabetes; future work should validate in Type 1 diabetes. Further functional studies are needed to clarify its role in diabetes.

## Supporting information

S1 Fig Full CIBERSORT analysis of 22 immune cell subtypes. (TIFF)

S2 Fig Diversity indices for BCR (IGH/IGK/IGL) and TCR (TRA/TRB/TRD/TRG) repertoires. No significant differences in BCR diversity.
(TIF)
