# Supplementary figures and images for: Immune repertoire profiling and T cell dysregulation in Peripheral Blood Mononuclear Cells of Type 2 diabetic patients
Source: PLoS One. 2025 Oct 9;20(10):e0332736. doi: 10.1371/journal.pone.0332736 (PMC12510570; doi:10.1371/journal.pone.0332736)

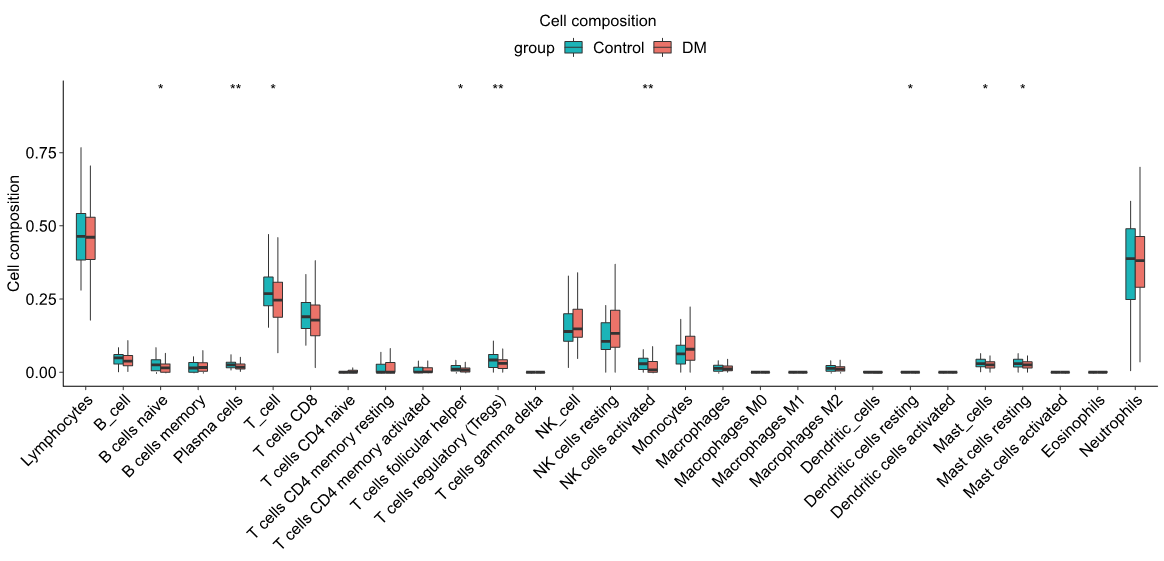

Supplement: S1 Fig — (TIFF) [file pone.0332736.s001.tiff]

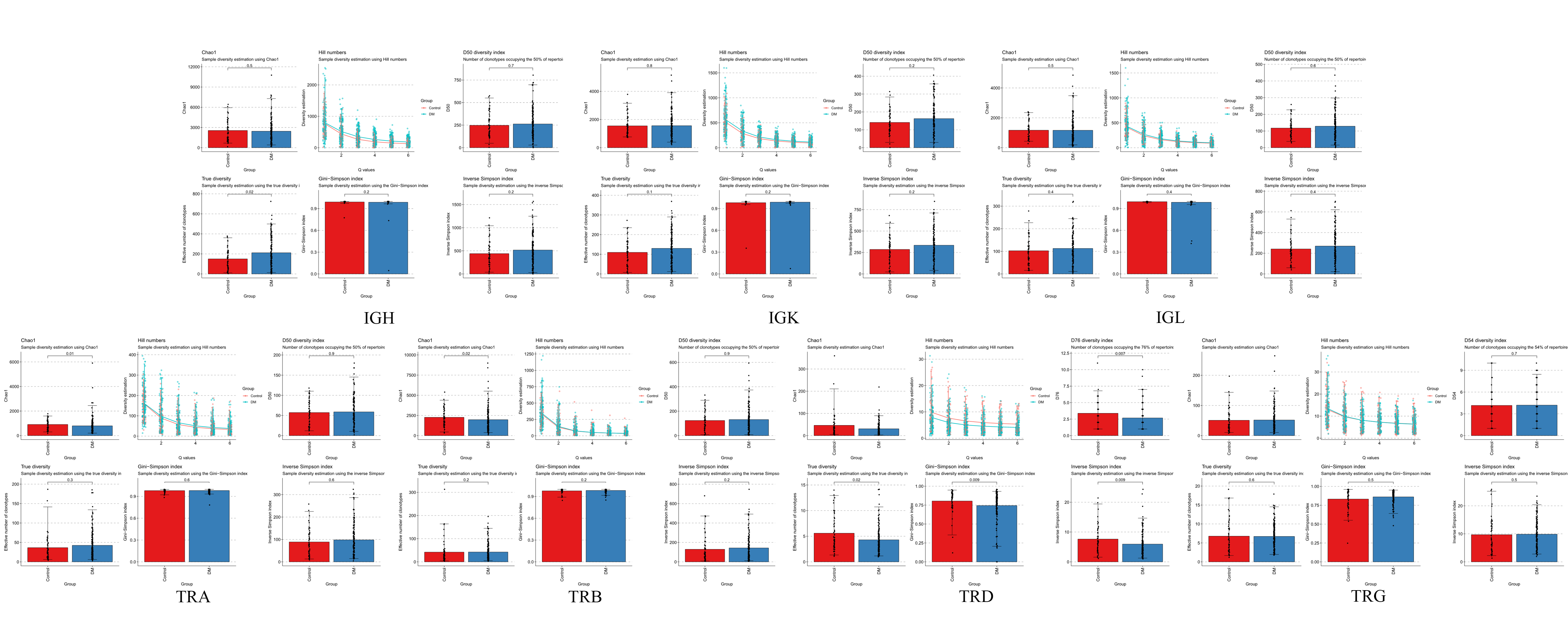

Supplement: S2 Fig — No significant differences in BCR diversity. (TIF) [file pone.0332736.s002.tif]
